# Association between 25-Hydroxyvitamin D, Parathyroid Hormone, Vitamin D and Calcium Intake, and Bone Density in Healthy Adult Women: A Cross-Sectional Analysis from the D-SOL Study

**DOI:** 10.3390/nu11061267

**Published:** 2019-06-04

**Authors:** Marcela M. Mendes, Kathryn H. Hart, Susan A. Lanham-New, Patrícia B. Botelho

**Affiliations:** 1 Department of Nutritional Sciences, University of Surrey, Guildford GU2 7XH, UK; k.hart@surrey.ac.uk (K.H.H.); s.lanham-new@surrey.ac.uk (S.A.L.-N.); 2 Postgraduate program of the Faculty of Nutrition, Federal University of Goiás, Goiânia 74605-080, Brazil; patriciaborges.nutri@gmail.com; 3 Faculty of Health Sciences, University of Brasília, Brasília 70910-900, Brazil

**Keywords:** 25-hydroxyvitamin D, parathyroid hormone, bone, vitamin D, Brazil, bone mineral density

## Abstract

There is still limited data on the association between 25-hydroxyvitamin D (25(OH)D), parathyroid hormone (PTH), and bone health in healthy younger adults, particularly in Latin America. This cross-sectional analysis aimed to investigate the associations of 25(OH)D and plasma PTH concentrations with bone parameters, and potential confounders, in women living in a high (England) or low (Brazil) latitude country. Bone was assessed by either peripheral quantitative computed tomography (pQCT) (England) or dual-energy x-ray absorptiometry (DXA) scan (Brazil), serum 25(OH)D concentrations by high performance liquid chromatography tandem mass spectrometry (HPLC-MS) and PTH by the chemiluminescent method. In participants living in England, total volumetric bone mineral density (vBMD) was significantly higher in women <29 years compared to ≥30 years, and total and cortical vBMD values at the 66% site were negatively correlated with weight and body mass index (BMI). In participants living in Brazil, age was positively correlated with bone mineral density (BMD) at the femur and bone mineral content (BMC), and weight, BMI, and body fat were correlated with BMD (lumbar spine and femur) and BMC. PTH concentrations were negatively correlated with 25(OH)D concentrations, and the prevalence of secondary hyperparathyroidism was 28.6% (*n* = 14) in participants with concentrations <25 nmol/L and 12.2% (*n* = 41) with concentrations between 25 and 49.9 nmol/L, compared to 6.3% (*n* = 79) in those with concentrations ≥50 nmol/L. In conclusion, weight and BMI were significantly correlated with bone parameters in both groups and age was significantly correlated with BMD at the femoral neck for women living in Brazil only. Although 25(OH)D concentrations were not correlated to bone parameters at any sites, in either country, PTH concentrations showed a significant correlation with total vBMD at the 66% site for women living in England. Secondary hyperparathyroidism was more common amongst those with deficient and insufficient vitamin D status.

## 1. Introduction

Calcium and vitamin D are essential nutrients with a major role in bone health. Calcium is the most abundant mineral in the body and is essential to functions related to vascular contraction, muscle function, nerve transmission, intracellular signaling, and hormonal secretion [1,2]. Bone acts as the calcium reservoir to maintain constant calcium homeostasis, with around 99% of the organism’s calcium supply stored in the bones and teeth. Vitamin D also plays a key role in calcium homeostasis, promoting calcium absorption in the gut and resorption in the kidney, as well as stimulating bone formation and remodeling [3]. Therefore, optimal intakes of calcium and vitamin D are generally regarded as fundamental factors in the prevention and treatment of osteoporosis.

The associations of calcium intake, vitamin D intake, and vitamin D status with bone mineral density (BMD) and the risk of fractures have been studied extensively over the past decades, but mostly in the elderly population [4,5,6]. An extensive amount of human evidence supports the contribution of calcium for bone homeostatic regulation and treatment of osteoporosis. Observational studies as well as randomized controlled trials have generally shown that higher calcium and vitamin D intakes (either from food or supplementation), compared to lower intakes, may have significant benefits to bone health and reduce the risk of osteoporosis [1,7,8,9].

Parathyroid hormone (PTH), a hormone secreted by the parathyroid glands in response to low serum calcium levels, is also well recognized as a fundamental part of bone homeostasis. PTH triggers the hydroxylation of 25-hydroxyvitamin D (25(OH)D) to the active form, 1,25-dihydroxyvitamin D (1,25(OH)_2_D), leading to enhanced intestinal absorption of calcium. Chronic elevated PTH concentrations can have significant negative impacts on BMD and consequently increase the risk of fractures over time [2,3,9]. Low dietary calcium and vitamin D intakes, as well as an inadequate vitamin D status, can be independent contributors to high PTH concentrations [10,11]. The negative correlation between PTH and vitamin D, and negative effects of higher PTH on bone health, have been previously demonstrated, but mostly in elderly and/or osteoporotic populations [11,12,13,14,15], and therefore there is still limited data on healthy adults, particularly in Latin America and Brazil [16].

This analysis aimed to investigate the associations of serum 25(OH)D and plasma PTH concentrations, as well as habitual dietary vitamin D and calcium intakes, age, and adiposity with bone health parameters assessed by either peripheral quantitative computed tomography (pQCT) or dual-energy x-ray absorptiometry (DXA) in in two groups of adult Brazilian women living in opposite latitudes.

## 2. Materials and Methods

A cross-sectional analysis of endocrine status (specifically 25(OH)D and PTH concentrations), calcium and vitamin D intake, age and adiposity, and bone mineral density was conducted in 130 healthy adult Brazilian women living in England (51° N) and healthy adult Brazilian women living in Brazil (16° S), recruited for the D-SOL study (Interaction between Vitamin D Supplementation and Sunlight Exposure in Women Living in Opposite Latitudes—clinicaltrials.gov as NCT03318029). The D-SOL study recruited adult women aged 20 to 59 years with Brazilian nationality (born in Brazil and having at least one parent born in Brazil).

### 2.1. Exclusion Criteria

The participants were screened according to exclusion criteria that included potential cofounders likely to affect vitamin D metabolism (osteoporosis therapy, anti-estrogen treatment, antiepileptic drugs, cancer treatment), taking containing vitamin D (if the prospective participants agreed to stop vitamin D supplementation to join the study, a wash-out period of 8 weeks prior to commencing the trial was accepted), being pregnant or planning a pregnancy during the study period, menopause status (defined as the permanent cessation of menstruation), and living in the UK for less than 3 months at the commencement of the study (for England participants only). All participants provided written informed consent at the commencement of the study.

### 2.2. Data Collection

The dietary intake of participants, particularly vitamin D and calcium, was determined by 4 consecutive days of estimated diet diaries, including one weekend day. Participants were instructed by the research team on how to correctly complete the diary and asked to give as much detail as possible of every meal, including portion size.

Dietary intake data obtained from participants in the England cohort were analyzed using the Nutritics^®^ nutritional analysis software (version 4.0, Dublin, Ireland), (UK Composition of Foods Integrated Dataset (CoFID) including McCance and Widdowson 7th edition (70)) and those collected in Brazil were analyzed via the Dietwin^®^ software (Version 13 (3090), Rio Grande do Sul, Brazil) (Brazilian Food Composition Table (TACO) (113) and the food composition database from the Brazilian Institute of Geography and Statistics (IBGE), as well as the United States Department of Agriculture (USDA) food composition database).

Participants were instructed to wear light clothing on the day of the visits to minimize different additional weights from clothing. For weight measurement, participants were asked to remove shoes, socks, and heavy coats before stepping on the scale. For the England trial, weight to the nearest 0.1 kg and body fat was obtained using a Tanita Body Composition Analyser MC-180MA (Tanita Cooperatives, Tokyo, Japan). For the Brazil trial, weight was measured to the nearest 0.1 kg using a standard weighing scale (Balmak^®^, Santa Bárbara d’Oeste, SP, Brazil) and body composition was determined via DXA scan (GE Healthcare LunarTM DPX NT + 152000, GE Medical Systems, Madison, WI, USA). Anthropometric methods were standardized between the different researchers involved in the study performing the measurements in each country to minimize inter-evaluator variations. Standing height was measured using a wall stadiometer to the nearest 0.1 cm, with participants in an upright posture and barefoot with heels close together and as close as possible to the wall. Waist circumference was measured with a non-extendable standard measure tape, at the narrowest point of the torso, to the nearest 0.1 cm. If this point could not be estimated, the level of the belly button was used as a reference point [17].

An overnight fasted (8 h) blood sample was collected by venipuncture by trained phlebotomists. Processed serum and plasma samples were divided into aliquots and stored at −80 °C at the University of Surrey, prior to analysis. Samples collected in Brazil followed the exact same procedures and were temporarily stored at −80 °C at the Federal University of Goiás, and sent to be stored at the University of Surrey as well, prior to analysis.

### 2.3. Laboratory Analysis

Serum 25(OH)D concentrations were determined by the HPLC-MS/MS method on a Waters Acuity UPLC (Triple Quadrupole) TQD^®^ System using a Pentafluorophenyl (PFP) column following supported liquid extraction (SLE). Laboratory intra- and inter-assay coefficients of variation (CV) were 5.6% and 7.8%, respectively. Calcium, albumin, and PTH concentrations were measured using Abbott Architect kits. Serum calcium was measured by using an endpoint spectrophotometric reaction based on the o-cresolphthalein complexone methodology, and serum albumin was measured by using an endpoint spectrophotometric reaction based on the bromocresol green solution dye binding methodology. Serum calcium concentrations were adjusted for albumin concentrations. Plasma intact PTH was measured by in vitro chemiluminescent microparticle immunoassay (CMIA). The manufacturer’s quoted inter-assay CV for calcium was <3%, for albumin <3.8%, and for PTH 4%.

### 2.4. Bone Measurements

For the England trial, a peripheral quantitative computed tomography scan (pQCT; XCT 2000, Stratec Medizintechnik GmbH, Pforzheim, Germany) was performed on the participant’s non-dominant forearm to measure volumetric bone mineral density at the 4% and 66% radial site. pQCT was performed by the same experienced operator to scan all participants’ radii. For total density and trabecular density, these were calculated using previously published reference data for white Caucasian European women [18] using the equation: Z-score = ((individual value − expected value for age)/reference standard deviation (SD)). For participants in Brazil, whole body bone mineral density, lower spine (L1–L4) density, and left femur bone mineral density were measured via DXA scan (dual-energy X-ray absorptiometry). The same experienced operator performed all DXA scans with all participants. Z-scores of Brazilian participants for the lumbar spine (L1–L4) and femur (femoral neck) were automatically calculated by the DXA scan, which used white Caucasian Women as a reference [19].

The same cut-off values for Z-score classification (i.e., Z-score ≥ −2.0 defined as normal (low-risk score) and Z-score ≤ −2.0 defined as low bone mineral for age) were applied to pQCT and DXA scans.

The study received a favorable ethical opinion from the University of Surrey (UEC/2016/009/FHMS) and Federal University of Goiás Ethics Committees and from the Brazilian National Ethics Committee (CONEP) (CAAE 62149516.9.0000.5083, CEP-UFG nº2013222; CONPEP nº 1972029; respectively).

### 2.5. Statistical Analysis

Statistical analysis of the data was done using SPSS software for Windows (version 25.0; IBM Corp, Armonk, NY, USA). Data were tested for normal distribution using the Kolmogorov–Smirnov tests. Non-normally distributed variables were log transformed and reported on the original scale. Non-parametric tests were used when log transforming did not normalize the data. Descriptive statistics were determined for all variables. Continuous variables are presented as mean ± standard deviation (SD) for normally distributed variables or as median (25%, 75% percentiles) for not normally distributed. For categorical variables, the frequency and percentage are reported.

Baseline characteristics (age, weight, BMI, waist circumference, dietary intakes, and biomarkers) were compared between countries, by independent t-tests, or Mann–Whitney U tests if appropriate.

For each country separately, ANOVA, or the corresponding non-parametric Kruskal–Wallis test, were used to compare bone parameter measurements between age tertiles and between vitamin D status groups. Pearson’s correlation, or the corresponding non-parametric Spearman rho, were applied to investigate the correlation between bone parameter measurements and 25(OH)D or PTH concentrations as well as vitamin D and calcium intakes.

Regression models were used to investigate the association between bone parameters and age and anthropometric measurements.

Results are presented separately for each country as measurements derived from different methodologies.

A *p* value of <0.05 was considered significant.

## 3. Results

### 3.1. Participant Characteristics

Baseline anthropometric, dietary, and biochemical characteristics are presented in Table 1, by country of residence. Brazilian women living in England were significantly older (*p* < 0.001), heavier (*p* = 0.002), and had a greater waist circumference (*p* < 0.001) than those living in Brazil. Amongst the 130 participants, 63% identified themselves as white and 33.3% as brown (mixed). The proportion of white women in the England cohort was higher (78.6% white compared to 1.8% black and 17.9% brown) while in the Brazil cohort there was an even distribution between white and brown (51.9% and 44.3%, respectively), with only 2.5% black.

Overall (women living in Brazil combined with those living in England, *n* = 114), mean habitual vitamin D dietary intake was 2.44 ± 1.91 μg/day and mean calcium intake was 627.053 ± 315.45 mg/day. Mean vitamin D and calcium intakes were significantly higher in England residents compared to Brazil residents (*p* < 0.001 and *p* = 0.003, respectively) (Table 1). In total (*n* = 114), 99.2% had vitamin D intakes below the recommended 10 μg/day [20], with only one participant recording intakes above this threshold. Only one participant living in Brazil was on vitamin D supplementation at the time of the first screening interview and suspended the supplementation two months prior to commencing the study in order to be enrolled. For calcium, 91.2% had average intakes below the Recommended Dietary Allowance (RDA) (US) [21] of 1000 mg/day for women ≤50 years, and 77.2% below the Reference Nutrient Intake (RNI) (UK) [22] of 700 mg/day for women >19 years.

In participants living in England, the mean 25(OH)D concentration was significantly lower and the PTH concentration was significantly higher than for participants in Brazil (Table 1). There were no significant differences between women living in England and in Brazil for serum calcium concentrations, which were within the normal range for all participants (Table 1).

### 3.2. Bone Density

Radial bone parameter measurements, determined by pQCT, for women living in England are shown in Table 2, stratified by the age tertiles. Total volumetric BMD at both distal and diaphyseal sites was significantly higher in women younger than 29 years of age compared to those 30 years or older (*p* < 0.001). There were no significant differences between age tertile groups in any other bone parameters. Only one participant in the England cohort (30–36 years old group) had a low Z-score (low bone mineral for age) for total volumetric BMD at the distal site. There were no significant differences between age tertile groups in weight, height, serum 25(OH)D, plasma PTH, nor serum calcium.

The characteristics of adult Brazilian women living in England divided by the tertiles of BMD can be found in the supplementary material (Tables S1–S4). Women in the lower tertile for trabecular vBMD at the 4% site were significantly taller than those in the middle tertile (*p* = 0.007). There were no significant differences in any other characteristics between BMD tertiles.

Lumbar spine (L1–L4) and femur measurements, determined by DXA, for women living in Brazil are shown in Table 3, stratified by age tertiles. The plasma PTH concentration was significantly lower in women younger than 25 years of age compared to those 31 years or older (*p* = 0.034). However, there were no significant differences between age groups in any of the bone parameters. Only two participants presented with low bone mineral values for age (Z-score ≤ −2.0) in the lumbar spine and both were younger than 25 years old.

The characteristics of adult Brazilian women living in Brazil divided by the tertiles of BMD can be found in the supplementary material (Tables S5 and S6). Women in the lower tertile for lumbar spine BMD had significantly lower weight than those in the highest tertile (*p* = 0.034). Women in the highest tertile for femur BMD had significantly higher weight and BMI values than those in the lowest and middle tertiles (*p* < 0.001 for both measures). There were no significant differences in any other characteristics between BMD tertiles.

### 3.3. Correlations between Bone Parameters and Age, Weight, and BMI

Among women living in England, the BMC at the distal site (4%) was positively correlated with weight and BMI (*p* = 0.021 and *p* = 0.024, respectively). Total vBMD at the diaphyseal site (66%) and cortical vBMD were negatively correlated with weight (*p* = 0.016 and *p* = 0.025, respectively) and BMI (*p* = 0.018 and *p* = 0.027, respectively) (Table 4). Body fat was not correlated with any of the bone parameters. When weight and BMI were entered into a regression model as predictors of BMC at the distal site, the model did not achieve statistical significance to explain the variation (*p* = 0.068). When weight and BMI were entered into a regression model as predictors of total vBMD at the diaphyseal site, 33.7% (*p* = 0.055) of the total variation was explained by the model, but none of the predictors had a significantly unique contribution. When weight and BMI were entered into a regression model as predictors of cortical vBMD, the model did not achieve statistical significance to explain the variation (*p* = 0.08).

In women living in Brazil, age was positively correlated with BMD at the femur and with BMC (*p* = 0.029 and *p* = 0.017, respectively), but not BMD at the lumbar spine (*p* = 0.292). Weight, BMI, and body fat were correlated with BMD at the lumbar spine and femur and with BMC (all *p* < 0.01). (Table 5). When age together with either weight, BMI, or body fat were entered into a regression model as predictors of BMD at the femur, the model explained 64.3% (*p* < 0.001) of the total variation, and only BMI had a significantly unique contribution (*p* = 0.027). When age together with weight, BMI, or body fat were entered into a regression model as predictors of BMC, the model explained 67% (*p* < 0.001) of the total variation, and only weight and BMI had significantly unique contributions to the model (*p* < 0.001 and *p* = 0.004, respectively). When weight, BMI, and body fat were entered into a regression model as predictors of BMD at the lumbar spine, 34.2% (*p* = 0.025) of the total variation was explained by the model, but none of the predictors had a significantly unique contribution.

### 3.4. Correlations between Bone Parameters and Habitual Dietary Vitamin D and Calcium Intakes

There were no significant correlations between radial bone parameter measurements, determined by pQCT, and habitual dietary vitamin D and calcium intakes for women living in England. Similarly, there were no significant correlations between lumbar spine (L1–L4) and femur bone parameter measurements, determined by DXA, and habitual dietary vitamin D and calcium intakes for women living in Brazil.

### 3.5. Correlations between Bone Parameters and 25(OH)D Concentrations

There was a trend for a weak positive relationship between total volumetric BMD at the diaphyseal site and 25(OH)D concentrations for women living in England, but significance was lost after controlling for age and BMI (*p* = 0.171). There were no significant correlations between any other bone parameter measurements. There were no significant correlations between lumbar spine (L1–L4) and femur bone parameter measurements, determined by DXA, and 25(OH)D concentrations for women living in Brazil.

When stratified by vitamin D status, women with vitamin D concentrations <25 nmol/L had significantly lower total (*p* = 0.0019) and cortical (*p* = 0.039) vBMD at the 66% site than women with concentrations between 25 and 49.9 nmol/L, and significantly lower total vBMD at the 66% site than women with concentrations above 50 nmol/L (0.044) (Table 6). After controlling for age and BMI, the significant differences remained only for total vBMD at the diaphyseal site, but not for cortical vBMD (ANCOVA *p* = 0.047 and *p* = 0.170, respectively).

There were no significant differences in any bone parameter measurements by vitamin D status for Brazilian women living in Brazil (Table 7).

### 3.6. Correlations between Bone Parameters and PTH Concentrations

**Figure 1:**
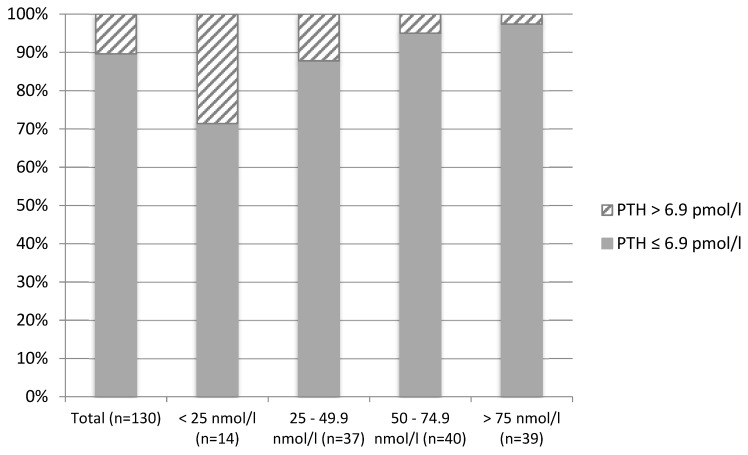
Prevalence of secondary hyperparathyroidism (Parathyroid Hormone > 6.9 pmol/L) according to vitamin D status in Brazilian women recruited to a vitamin D supplementation study (*n* = 130).

**Figure 2:**
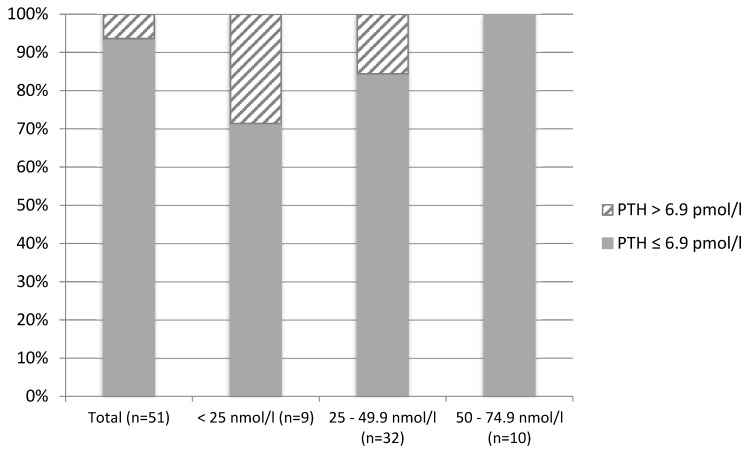
Prevalence of secondary hyperparathyroidism (PTH > 6.9 pmol/L) according to vitamin D status in Brazilian women living in England (*n* = 51).

**Figure 3:**
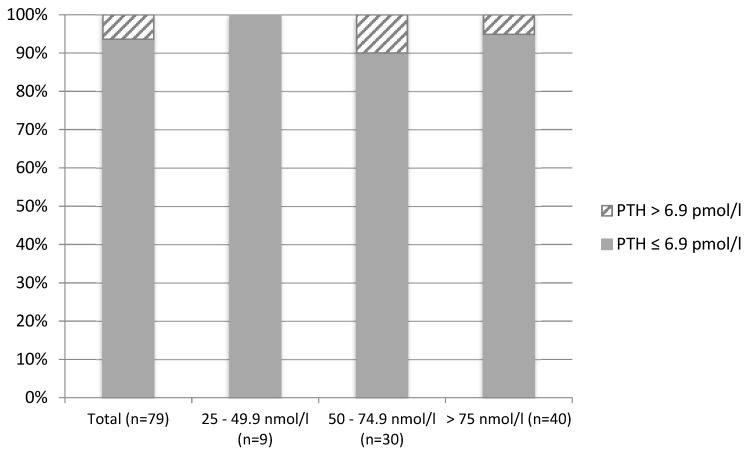
Prevalence of secondary hyperparathyroidism (PTH > 6.9 pmol/L) according to vitamin D status in Brazilian women living in Brazil (*n* = 79).

Overall (women living in Brazil combined with those living in England, *n* = 130), plasma PTH concentrations were negatively correlated with 25(OH)D concentrations (r = −0.285, *p* = 0.001). Within each group, plasma PTH concentrations were also negatively correlated with 25(OH)D concentrations in women living in the UK (r = −0.286, *p* = 0.042), but not in those living in Brazil (r = −0.113, *p* = 0.323). In total (*n* = 130), 10.4% of participants presented with secondary hyperparathyroidism (PTH concentrations > 6.9 pmol/L). The prevalence of secondary hyperparathyroidism according to vitamin D status was 28.6% among participants with concentrations <25 nmol/L (*n* = 14) and 12.2% within participants with concentrations between 25 and 49.9 nmol/L (*n* = 41). Only 3 participants with concentrations between 50 and 74.9 nmol/L and 2 participants with concentrations >75 nmol/L presented with secondary hyperparathyroidism (*p* = 0.086 for comparison of prevalence across status groups) (Figure 1). When analyzed separately for the two groups of women, the difference in the prevalence of secondary hyperparathyroidism was more evident between the two lower cut-off points for women living in England (Figure 2) and between the two highest cut-off points for women living in Brazil (Figure 3).

Correlations between radial bone parameter measurements, determined by pQCT, and PTH concentrations for women living in England are shown in Table 8. There was a significant positive association between PTH concentrations and BMC at both the 4% and 66% sites (*p* = 0.0012 and *p* = 0.001, respectively). Total vBMD at the 66% site was significantly negatively correlated with PTH concentrations (*p* = 0.026). However, after controlling for age and BMI, significance remained only for BMC at the diaphyseal site (*p* = 0.039), but not for BMC at the distal site or total vBMD at the diaphyseal site (*p* = 0.064 and *p* = 0.078, respectively).

There were no significant correlations between lumbar spine (L1–L4) and femur bone parameter measurements, determined by DXA, and PTH concentrations for women living in Brazil (Table 9).

## 4. Discussion

Vitamin D is essential for musculoskeletal health, and there is a consensus that serum concentrations should be at least 25 nmol/L to prevent detrimental effects on bone [1,2,3]. The present cross-sectional analysis of healthy adult women showed that amongst Brazilian women living in England, all participants presented a normal radial z-score. Amongst women living in Brazil, only two participants (2.5%) presented with low bone mineral for age for the lumbar spine. Furthermore, bone parameters were significantly associated with weight and BMI in participants in both the England and Brazil cohorts, and with age in women living in Brazil only. This study also found that for women living in England, those with vitamin D deficiency (<25 nmol/L) had significantly lower total vBMD at the 66% site than those with greater concentrations, independent of age and BMI. This observation at the cortical site (66%) only is likely due to the fact that most bone loss is the result of intracortical and endocortical remodeling, which produces cortical porosity and cortical thinning. Therefore, any alteration of BMD would be first evidenced at the cortical (66%) in comparison to the trabecular (4%) site. There were no associations between 25(OH)D status and any of the measures from the DXA scan in participants from the Brazil cohort, although the range of vitamin D status was smaller and the generally better vitamin D status of this sub-group may have limited the observation of effects on the bone. Nevertheless, the finding of bone parameters being significantly associated with the 25(OH)D/PTH system in women living in England, but not in those living in Brazil, together with the finding that plasma PTH concentrations were negatively correlated with 25(OH)D concentrations in women living in the UK, but not in those living in Brazil, are relevant for speculating on the possible role of different lifestyles and climates in mediating the relationship between 25(OH)D, PTH, and bone.

The associations between bone parameters and weight and BMI suggest that the observed poorer bone health in this sample is likely to be due to lower weights. These findings are in agreement with several studies that have demonstrated positive associations between BMI and bone parameters, likely explained by a greater load on weight-bearing bones [23,24,25]. In a cross-sectional study investigating a total of 412 Brazilian postmenopausal women, aged 40 to 75 years with BMD assessed by DXA at the lumbar spine, a higher BMI was associated with reduced osteoporosis risk [26]. A study with 393 post-menopausal Brazilian women reported a lower prevalence of osteopenia and osteoporosis amongst obese women compared to those with eutrophic BMI (DXA-derived BMD assessment) [27]. Another study in São Paulo, with 413 Brazilian women (52.5% <59 years and 47.5% >60 years) showed the BMI to be a positive predictor for DXA-derived BMD at the femoral neck [28]. For women living in England, there was an inverse statistically significant correlation between vBMD at the 66% site (total and cortical) and weight. Some studies have reported similar findings of the inverse correlation in the cortical site of the radius observed in this study [29,30]. Some hypotheses to explain this include variances in bone geometry or bone damage, increased bone turnover, and a momentary reduction in vBMD due to increased mechanical loading. Further investigation is now required, especially in adult women under 50 years of age, to better understand these relationships [29,31,32].

To date, there are still few longitudinal studies that have investigated the association of long-term lower calcium intake with bone health outcomes later in life, particularly from a younger starting age, i.e., <30–35 years. A longitudinal study of 5022 women (born between 1914 and 1948 and followed up for 19 years) with modest dietary calcium intake reported that only the lowest quintile of calcium intake (<751 mg) was associated with increased risk of fracture or osteoporosis [33]. It has been suggested that the association between calcium and BMD might not be consistently linear, and a sufficient vitamin D status is likely to compensate for the negative effects of low calcium intake on bone [34,35,36].

In the United Kingdom, the latest national survey reported mean dietary calcium intakes of 897 and 746 mg/day for men and women aged 19 to 64 years, respectively (NDNS years 7–8, 2015–2016). In Brazil, the latest national survey available to date reported that mean dietary calcium intakes were 546.4 and 476.4 mg/day for men and women aged 20 to 59 years, respectively (POF, 2008–2009) [37]. In this study, 72.8% of participants had dietary calcium intakes below the RNI of 700 mg/day, while only 5.2% met the 1000 mg/day RDA reference. All participants had dietary vitamin D intakes below the 15 μg/day RDA recommended by the IOM [21] and 99.2% had intakes below the RNI of 10 μg/day proposed by SACN [20]. Despite this, there were no significant correlations between bone parameter measurements and habitual dietary vitamin D and calcium intakes, either for England or Brazil dwelling participants, perhaps again reflecting the narrow range of intakes and therefore lack of discriminatory power in this sample.

Elevated concentrations of serum PTH are associated with several adverse outcomes, particularly musculoskeletal outcomes [38,39,40]. Secondary hyperparathyroidism may lead to bone loss due to increased bone turnover rates [38,41]. Several studies have shown 25(OH)D to be inversely correlated with PTH [10,38,40,42]. The present study confirms this with 25(OH)D concentrations being inversely correlated with PTH concentrations in healthy adult women. Additionally, 10.4% of participants had secondary hyperparathyroidism, with a higher prevalence amongst those with deficient and insufficient vitamin D status. The difference in the prevalence of secondary hyperparathyroidism was more evident between the two lower cut-off points for women living in England and between the two highest cut-off points for women living in Brazil, which might be explained by the significant difference in the mean and range of 25(OHD concentrations between the two groups. Due to the strong inverse correlation between 25(OH)D and PTH concentrations, individuals with deficient and insufficient vitamin D status would very likely benefit from vitamin D supplementation with a view to suppressing PTH secretion from the parathyroid gland.

Moreover, several studies have also shown an inverse correlation between serum PTH levels and BMD [40,41]. In this study, a negative correlation between PTH concentrations and total BMD at the 66% site was seen, although this was not significant after controlling for age and BMI. There were no associations with PTH levels and any of the measures from the DXA scan in participants from the Brazil cohort, which may be due to long-term detrimental consequences of higher PTH levels not yet being evident in the relatively young women in this study.

The associations between 25(OH)D, PTH, and bone mineral density (BMD) are still much debated. Evidence is more robust in specific subgroups, such as in those with low vitamin D levels [39], in osteoporotic subjects [9,11], post-menopausal women [9,11,43,44], or in the elderly [14,38,45]. The findings presented here are in accordance with previous reports of studies that included healthy younger adults as well as those that encompass the whole spectrum of vitamin D status (i.e., deficient, sufficient, and adequate), demonstrating the absence of any association between 25(OH)D or PTH and BMD [13,46,47]. The reasons for no effects on bone parameters might be due to the lack of power within the deficient subgroup and the relatively young participants, who might not be currently affected by potential long-term detrimental outcomes from low 25(OH)D and high PTH concentrations. Further studies are now required to better understand the effects of inadequate concentrations of 25(OH)D or PTH on bone health specifically in younger otherwise healthy women and future health consequences.

The strengths of this study include the fact that both measurements used for bone density assessment, pQCT and DXA scan, are considered gold standard methods. The analysis controlled for possible confounders, such as age, weight, and BMI. A limitation of the study is that the DXA scan software reports using a combination of databases to calculate the Z-score, while for the pQCT data a specific published reference data was used. Ideally, the same method would have been used for both groups, and a DXA scan would be the preferred option, as it has been shown to be an accurate diagnostic clinical tool and would have provided a central measurement for all participants [48]. It might be that there was insufficient statistical power in this sample to detect significant differences in bone parameters and serum 25(OH)D and PTH concentrations. This analysis would benefit from further measures of biochemical markers of bone turnover to further investigate the relationship of 25(OH)D, PTH, and bone health in these adult women.

In conclusion, weight and BMI were significantly correlated with bone parameters in both groups and age was significantly correlated with BMD at the femoral neck for women living in Brazil only. Although 25(OH)D concentrations were not correlated to bone parameters at any sites, in either country, PTH concentrations showed a significant correlation with total vBMD at the 66% site for women living in England. This analysis also showed that secondary hyperparathyroidism was more common amongst those with a deficient and insufficient vitamin D status. There were no significant correlations between bone parameters and the usual dietary intake of vitamin D and calcium, in either of the groups.

## Figures and Tables

**Table 1:** Characteristics of adult Brazilian women by country of residence (*n* = 130) ^1^.

	England (*n* = 51)	Brazil (*n* = 79)	*p* Value ^2^
Age (years)	33 (28, 38)	27 (24, 31)	<0.001
Height (m)	1.64 ± 0.05	1.62 ± 0.59	0.094
Weight (kg)	70.11 ± 14.22	63.21 ± 13.07	0.002
Waist Circumference (cm)	86.3 (77.0, 96.0)	70.4 (66.1, 77.2)	<0.001
BMI (kg/m^2^)	25.94 ± 5.30	24.01 ± 4.88	0.009
Body fat (%) ^≠^	30.95 ± 6.00	38.65 ± 8.52	<0.001 ^a^
Vitamin D intake (μg/day)	2.59 (1.55, 3.92)	1.57 (0.73, 2.79)	<0.001
Calcium intake (mg/day)	711.76 (537.22, 879.51)	479.97 (347.42, 704.92)	0.003
Serum 25(OH)D (nmol/L)	35.22 ± 14.89	75.00 ± 22.13	<0.001 ^a^
Plasma PTH (pmol/L)	5.39 ± 2.07	4.49 ± 1.47	0.004 ^a^
Serum Calcium (mmol/L) ^3^	2.30 ± 0.07	2.28 ± 0.06	0.066 ^a^

^1^ Values are median (25th, 75th percentile) or mean ± SD. ^2^ Statistical analysis: Mann–Whitney U unless otherwise stated; ^a^ Independent *t*-test. ^3^ Albumin corrected serum calcium concentrations. ^≠^ Measurements derived from different methodologies (England: bio-impedance; Brazil: DXA scan).

**Table 2:** Radial bone densitometry assessed by peripheral Quantitative Computed Tomography (pQCT) of Brazilian women living in England by age tertiles (*n* = 51) ^1^.

Bone Parameter	All	Age			*p* Value ^2^
		Tertile 1	Tertile 2	Tertile 3	
		(≤29 Years)	(30–36 Years)	(>37 Years)	
Number (n)	51	18	16	17	
Wegiht (kg)	70.11 ± 14.22	64.70 ± 11.24	72.71 ± 15.68	73.38 ± 14.69	0.132
Height (m)	1.64 ± 0.05	1.63 ± 0.05	1.65 ± 0.06	1.64 ± 0.04	0.518
Serum 25(OH)D (nmol/L)	35.22 ± 14.89	36.78 ± 13.20	33.74 ± 15.52	34.95 ± 16.64	0.84
Plasma PTH (pmol/L)	5.39 ± 2.07	5.31 ± 2.00	5.29 ± 2.54	5.56 ± 1.74	0.92
Serum calcium (mmol/L)	2.30 ± 0.07	2.31 ± 0.08	2.28 ± 0.06	2.29 ± 0.07	0.388
4% site					
BMC (g/cm)	1.07 ± 0.14	1.05 ± 0.15	1.08 ± 0.13	1.08 ± 0.13	0.821
vBMD (mg/cm^3^):					
Total	353.20± 51.81	380.72 ± 60.82 ^a^	329.97 ± 37.07 ^a^	346.12 ± 41.47	0.011
Trabecular	199.29 ± 57.78	205.61 ± 73.89	189.48 ± 44.14	201.85 ± 51.53	0.709
66% site					
BMC (g/cm)	0.96 ± 0.14	0.93 ± 0.15	0.97 ± 0.12	0.97 ± 0.14	0.557
vBMD (mg/cm^3^):					
Total	714.38 ±86.33	753.33 ± 68.57	693.10 ± 90.80	693.17 ± 89.02	0.056
Cortical	1127.23 ± 36.44	1140.09 ± 27.03	1124.26 ± 37.61	1116.43 ± 41.59	0.147
Z-score n (%)					
Total vBMD (4%)					
Normal (>–2.0)	51 (100%)	18 (100%)	16 (100%)	17 (100%)	
Low bone mineral (≤–2.0)	0	0	0	0	
Trabecular vBMD (4%)					
Normal (>–2.0)	51 (100%)	18 (100%)	16 (100%)	17 (100%)	
Low bone mineral (≤–2.0)	0	0	0	0	
Total vBMD (66%)					
Normal (>–2.0)	48 (94.2%)	18 (100%)	13 (94.8%)	17 (100%)	
Low bone mineral (≤–2.0)	3 (5.8%)	0	3 (18.7%)	0	
Cortical vBMD (66%)					
Normal (>−1.0)	51 (100%)	18 (100%)	16 (100%)	17 (100%)	
Low bone mineral (≤−2.0)	0	0	0	0	

^1^ Values: mean ± SD or n (%); ^2^ Statistical analysis: one-way ANOVA with post-hoc Tukey’s test. Values in the same row with the same superscript letters are significantly different (^a^ *p* < 0.001).

**Table 3:** Lumbar spine and femur bone densitometry assessed by Dual-energy X-ray absorptiometry (DXA) of Brazilian women living in Brazil by age tertiles (*n* = 79) ^1^.

Bone Parameter	All	Age (Years)			*p* Value ^2^
		Tertile 1 ≤25	Tertile 2 26–30	Tertile 3 ≥31	
Number (n)	79	33	25	21	
Weight (kg)	63.21 ± 13.07	60.41 ± 12.58	64.30 ± 12.46	66.32 ± 14.20	0.239
Height (m)	1.62 ± 0.59	1.61 ± 0.05	1.64 ± 0.06	1.60 ± 0.06	0.157
Serum 25(OH)D (nmol/L)	75.00 ± 22.13	78.87 ± 27.23	74.68 ± 18.39	77.15 ± 17.61	0.868
Plasma PTH (pmol/L)	4.49 ±1.47	3.96 ± 1.25 ^a^	4.79 ± 1.61	4.99 ± 1.42 ^a^	0.021
Serum calcium (mmol/L)	2.28 ± 0.06	2.29 ± 0.07	2.29 ± 0.05	2.25 ± 0.07	0.26
Lumbar Spine					
Number (n)	79	33	25	21	0.537
BMD (g/cm^2^)	1.13 ± 0.11	1.12 ± 0.12	1.13 ± 0.11	1.15 ± 0.11	
Femur					
Number (n)	64	27	20	17	0.495
BMD (g/cm^2^)	0.98 ± 0.10	0.97 ± 0.10	0.97 ± 0.12	1.01 ± 0.11	
Total BMC					
Number (n)	79	33	25	21	0.204
BMC (g)	2304.87 ± 401.76	2304.87 ± 401.76	2459.92 ± 377.29	2460.95 ± 338.42	
Z-score n (%)					
Lumbar Spine BMD					
Normal (>−2.0)	79 (97.5%)	31 (94%)	25 (100%)	21 (100%)	
Low bone mineral (≤−2.0)	2 (2.5%)	2 (6%)	0	0	
Z-score n (%)					
Femur BMD					
Normal (>−2.0)	64 (100%)	27 (100%)	20 (100%)	17 (100%)	
Low bone mineral (≤−2.0)	0	0	0	0	

^1^ Values: mean ± SD or n (%); ^2^ Statistical analysis: one-way ANOVA with post-hoc Tukey’s test: values in the same row with the same superscript letters are significantly different (^a^ *p* = 0.034).

**Table 4:** Correlations between age and adiposity and radial bone densitometry assessed by pQCT of Brazilian women living in England by age tertiles (*n* = 51) ^1^.

Bone Parameter	Age (Years)		Weight (kg)		BMI (kg/m^2^)		Body Fat (%)	
	r Value	*p* Value	r Value	*p* Value	r Value	*p* Value	r Value	*p* Value
4% site								
BMC (g/cm)	0.186	0.191	0.323	0.021	0.317	0.024	0.118	0.386
vBMD (mg/cm^3^):								
Total	−0.143	0.316	−0.005	0.97	−0.028	0.847	0.034	0.814
Trabecular	−0.007	0.962	−0.118	0.41	−0.112	0.435	0.085	0.551
66% site								
BMC (g/cm)	0.216	0.128	0.256	0.07	0.253	0.074	−0.063	0.345
vBMD (mg/cm^3^):								
Total	−0.213	0.134	−0.335	0.016	−0.330	0.018	−0.159	0.266
Cortical	−0.223	0.116	−0.313	0.025	−0.309	0.027	−0.122	0.393

^1^ Pearson’s correlation.

**Table 5:** Correlations between age and adiposity and lumbar spine and femur bone densitometry assessed by DXA of Brazilian women living in Brazil by age tertiles (*n* = 79) ^1^.

Bone Parameter	Age (Years)		Weight (kg)		BMI (kg/m^2^)		Body Fat (%)	
	r Value	*p* Value	r Value	*p* Value	r Value	*p* Value	r Value	*p* Value
Lumbar Spine BMD (mg/cm^2^)	0.120	0.292	0.337	0.002	0.298	0.008	0.252	0.025
Femur BMD (mg/cm^2^)	0.273	0.029	0.583	<0.001	0.612	<0.001	0.378	0.002
Total BMC (g)	0.267	0.017	0.606	<0.001	0.585	<0.001	0.412	<0.001

^1^ Pearson’s correlation.

**Table 6:** Radial bone densitometry assessed by pQCT of Brazilian women living in England by vitamin D status (*n* = 51) ^1^.

Bone Parameter	All	25(OH)D in nmol/L			*p* Value ^2^
		<25	25–49.99	50–74.99	
Number (n)	51	14	28	9	
Weight (kg)	70.11 ± 14.22	76.01 ± 17.72	66.44 ± 12.6	72.33 ± 10.20	0.104
Height (m)	1.64 ± 0.05	1.63 ± 0.04	1.64 ± 0.05	1.67 ± 0.05	0.340
BMI (kg/m^2^)	25.94 ± 5.30	28.28 ± 6.13	24.73 ± 4.84	26.09 ± 4.54	0.123
Serum 25(OH)D (nmol/L)	35.22 ± 14.89	20.45 ± 5.33	34.29 ± 5.69	61.07 ± 9.74	>0.001
Plasma PTH (pmol/L)	5.39 ± 2.07	6.60 ± 2.47	5.00 ± 1.92	4.69 ± 0.92	0.030
Serum calcium (mmol/L)	2.30 ± 0.07	2.28 ± 0.05	2.29 ± 0.08	2.31 ± 0.05	0.634
4% site					
BMC (g/cm)	1.07 ± 0.14	1.06 ± 0.12	1.07 ± 0.15	1.10 ± 0.13	0.782
vBMD (mg/cm^3^):					
Total	352.23 ± 51.81	355.81 ± 40.47	356.86 ± 57.76	337.95 ± 50.35	0.630
Trabecular	199.29 ± 57.78	189.56 ± 25.61	208.46 ± 63.39	185.91 ± 57.87	0.462
66% site					
BMC (g/cm)	0.96 ± 0.14	0.97 ± 0.13	0.96 ± 0.16	0.92 ± 0.05	0.642
vBMD (mg/cm^3^):					
Total	714.39 ± 86.33	658.54 ± 104.23 ^a,b^	732.91 ± 73.85 ^a^	743.62 ± 53.80 ^b^	0.014
Cortical	1127.23 ± 36.44	1107.74 ± 44.43 ^c^	1136.66 ± 31.41 ^c^	1128.24 ± 28.01	0.049

^1^ Values: mean ± SD. ^2^ Statistical analysis: one-way ANOVA with post-hoc Tukey’s test. Values in the same row with the same superscript letters are significantly different (^a^ *p* = 0.019, ^b^ *p* = 0.044, ^c^ *p* = 0.039).

**Table 7:** Lumbar spine and femur bone densitometry assessed by DXA of Brazilian women living in Brazil by vitamin D status (*n* = 79) ^1^.

Bone Parameter	All	25(OH)D in nmol/L			*p* Value ^2^
		25–49.99	50–74.99	>75	
Weight (kg)	63.21 ± 13.07	57.49 ± 10.73	62.51 ± 12.24	65.03 ± 13.97	0.278
Height (m)	1.62 ± 0.59	1.60 ± 0.06	1.62 ± 0.06	1.62 ± 0.05	0.625
BMI (kg/m^2^)	24.01 ± 4.88	22.4 ± 3.09	23.65 ± 4.71	24.64 ± 5.29	0.409
Serum 25(OH)D (nmol/L)	75.00 ± 22.13	42.77 ± 4.12	61.86 ± 7.05	92.11 ± 16.31	>0.001
Plasma PTH (pmol/L)	4.49 ± 1.47	4.73 ± 1.23	4.50 ± 1.53	4.42 ± 1.51	0.86
Serum calcium (mmol/L)	2.28 ± 0.06	2.28 ± 0.06	2.28 ± 0.07	2.28 ± 0.06	0.993
Lumbar Spine					
Number (n)	79	9	30	40	0.716
BMD (mg/cm^2^)	1.13 ± 0.11	1.11 ± 0.11	1.13 ± 0.12	1.14 ± 0.10	
Femur					
Number (n)	64	8	25	31	0.728
BMD (mg/cm^2^)	0.98 ± 0.10	0.94 ± 0.10	0.97 ± 0.10	1.00 ± 0.11	
Total					
Number (n)	79	9	30	40	0.335
BMC (g)	2395.43 ± 381.24	2305.66 ± 372.97	2391.46 ± 448.47	2418.60 ± 331.93	

^1^ Values: mean ± SD. ^2^ Statistical analysis: one-way ANOVA with post-hoc Tukey’s test.

**Table 8:** Associations between PTH concentrations and radial bone densitometry assessed by pQCT of Brazilian women living in England (*n* = 51) ^1^.

Bone Parameter	PTH	
	r Value	*p* Value
4% site		
BMC (g/cm)	0.348	0.012
Total vBMD (mg/cm^3^)	0.108	0.45
Trabecular vBMD (mg/cm^3^)	0.085	0.553
66% site		
BMC (g/cm)	0.435	0.001
Total vBMD (mg/cm^3^)	−0.312	0.026
Cortical vBMD (mg/cm^3^)	−0.184	0.197

^1^ Pearson’s correlation.

**Table 9:** Correlations between PTH concentrations and lumbar spine and femur bone densitometry assessed by DXA of Brazilian women living in Brazil (*n* = 78) ^1^.

Bone Parameter	PTH	
	r Value	*p* Value
Lumbar Spine BMD (mg/cm^2^) (*n* = 78)	0.004	0.976
Femur BMD (mg/cm^2^) (*n* = 63)	0.215	0.900
Total BMC (g) (*n* = 78)	0.114	0.319

^1^ Pearson’s correlation.

## Supplementary Materials

The following are available online at https://www.mdpi.com/2072-6643/11/6/1267/s1, Table S1: Characteristics of Brazilian women living in England by tertile of 4% Total vBMD (*n* = 51); Table S2: Characteristics of Brazilian women living in England by tertile of 4% Trabecular vBMD (*n* = 51); Table S3: Characteristics of Brazilian women living in England by tertile of 66% Cortical vBMD (*n* = 51); Table S4: Characteristics of Brazilian women living in England by tertile of 66% Total vBMD (*n* = 51); Table S5: Characteristics of Brazilian women living in Brazil by tertile of lumbar spine BMD (*n* = 79); Table S6: Characteristics of Brazilian women living in Brazil by tertile of femur BMD (*n* = 64).

## References

[1] Heaney R., Marcus R., Dempster D., Cauley J., Feldman D. (2013). Calcium in the treatment of osteoporosis. Osteoporosis: Fourth Edition.

[2] Peacock M. (2010). Calcium metabolism in health and disease. Clin. J. Am. Soc. Nephrol..

[3] DeLuca H.F. (2004). Overview of general physiologic features and functions of vitamin D. Am. J. Clin. Nutr..

[4] Reid I.R., Bolland M.J., Grey A. (2014). Effects of vitamin D supplements on bone mineral density: A systematic review and meta-analysis. Lancet.

[5] Tang B.M., Eslick G.D., Nowson C., Smith C., Bensoussan A. (2007). Use of calcium or calcium in combination with vitamin D supplementation to prevent fractures and bone loss in people aged 50 years and older: A meta-analysis. Lancet.

[6] Silk L.N., Greene D.A., Baker M.K. (2015). The effect of calcium or calcium and vitamin D supplementation on bone mineral density in healthy males: A systematic review and meta-analysis. Int. J. Sport Nutr. Exerc. Metab..

[7] Jackson R.D., LaCroix A.Z., Gass M., Wallace R.B., Robbins J., Lewis C.E., Bassford T., Beresford S.A., Black H.R., Blanchette P. (2006). Calcium plus vitamin D supplementation and the risk of fractures. N. Engl. J. Med..

[8] Chapuy M.C., Pamphile R., Paris E., Kempf C., Schlichting M., Arnaud S., Garnero P., Meunier P.J. (2002). Combined calcium and vitamin D3 supplementation in elderly women: Confirmation of reversal of secondary hyperparathyroidism and hip fracture risk: The decalyos ii study. Osteoporos. Int..

[9] Dawson-Hughes B., Harris S.S., Krall E.A., Dallal G.E. (1997). Effect of calcium and vitamin D supplementation on bone density in men and women 65 years of age or older. New Engl. J. Med..

[10] Vieth R., Ladak Y., Walfish P.G. (2003). Age-related changes in the 25-hydroxyvitamin D versus parathyroid hormone relationship suggest a different reason why older adults require more vitamin D. J. Clin. Endocr. Metab..

[11] Lips P., Duong T., Oleksik A., Black D., Cummings S., Cox D., Nickelsen T., Ste-Marie L.G., Evalu M.O.R. (2001). A global study of vitamin D status and parathyroid function in postmenopausal women with osteoporosis: Baseline data from the multiple outcomes of raloxifene evaluation clinical trial. J. Clin. Endocr. Metab..

[12] Saraiva G.L., Cendoroglo M.S., Ramos L.R., Araujo L.M., Vieira J.G., Maeda S.S., Borba V.Z., Kunii I., Hayashi L.F., Lazaretti-Castro M. (2007). Prevalence of vitamin D deficiency, insufficiency and secondary hyperparathyroidism in the elderly inpatients and living in the community of the city of Sao Paulo, Brazil. Arq. Bras. Endocrinol. Metabol..

[13] Moreno-Reyes R., Carpentier Y.A., Boelaert M., El Moumni K., Dufourny G., Bazelmans C., Leveque A., Gervy C., Goldman S. (2009). Vitamin D deficiency and hyperparathyroidism in relation to ethnicity: A cross-sectional survey in healthy adults. Eur. J. Nutr..

[14] Melin A.L., Wilske J., Ringertz H., Saaf M. (1999). Vitamin D status, parathyroid function and femoral bone density in an elderly swedish population living at home. Aging Clin. Exp. Res..

[15] Russo L.A., Gregorio L.H., Lacativa P.G., Marinheiro L.P. (2009). Concentration of 25-hydroxyvitamin D in postmenopausal women with low bone mineral density. Arq. Bras. Endocrinol. Metabol..

[16] Chen T.C., Chimeh F., Lu Z.R., Mathieu J., Person K.S., Zhang A.Q., Kohn N., Martinello S., Berkowitz R., Holick M.F. (2007). Factors that influence the cutaneous synthesis and dietary sources of vitamin D. Arch. Biochem. Biophys..

[17] Marfell-Jones M.J., Stewart A.D., De Ridder J.H. (2012). International Standards for Anthropometric Assessment.

[18] Burt L.A., Macdonald H.M., Hanley D.A., Boyd S.K. (2014). Bone microarchitecture and strength of the radius and tibia in a reference population of young adults: An hr-pqct study. Arch. Osteoporos..

[19] Looker A.C., Wahner H.W., Dunn W.L., Calvo M.S., Harris T.B., Heyse S.P., Johnston C.C., Lindsay R. (1998). Updated data on proximal femur bone mineral levels of us adults. Osteoporos. Int..

[20] Scientific Advisory Committee on Nutrition (SCAN) (2016). Vitamin D and Health.

[21] Ross A.C., Manson J.E., Abrams S.A., Aloia J.F., Brannon P.M., Clinton S.K., Durazo-Arvizu R.A., Gallagher J.C., Gallo R.L., Jones G. (2011). The 2011 report on dietary reference intakes for calcium and vitamin D from the institute of medicine: What clinicians need to know. J. Clin. Endocrinol. Metab..

[22] Public Health England (2016). Government recommendations for energy and nutrients for males and females aged 1–18 years and 19+ years. Government Dietary Recommendations.

[23] Felson D.T., Zhang Y., Hannan M.T., Anderson J.J. (1993). Effects of weight and body mass index on bone mineral density in men and women: The framingham study. J. Bone Miner. Res..

[24] Johansson H., Kanis J.A., Oden A., McCloskey E., Chapurlat R.D., Christiansen C., Cummings S.R., Diez-Perez A., Eisman J.A., Fujiwara S. (2014). A meta-analysis of the association of fracture risk and body mass index in women. J. Bone Miner. Res..

[25] De Laet C., Kanis J.A., Oden A., Johanson H., Johnell O., Delmas P., Eisman J.A., Kroger H., Fujiwara S., Garnero P. (2005). Body mass index as a predictor of fracture risk: A meta-analysis. Osteoporosis. Int..

[26] Nahas E.A.P., Kawakami M.S., Nahas-Neto J., Buttros D.D., Cangussu L., Rodrigues A.B. (2011). Assessment of risk factors for low bone mineral density in brazilian postmenopausal women. Climacteric.

[27] Mazocco L., Chagas P. (2017). Association between body mass index and osteoporosis in women from northwestern Rio Grande do Sul. Rev. Bras. Reumatol..

[28] Frazao P., Naveira M. (2007). Factors associated with low bone mineral density among white women. Rev. Saude Publica.

[29] Barbour K.E., Zmuda J.M., Strotmeyer E.S., Horwitz M.J., Boudreau R., Evans R.W., Ensrud K.E., Petit M.A., Gordon C.L., Cauley J.A. (2010). Correlates of trabecular and cortical volumetric bone mineral density of the radius and tibia in older men: The Osteoporotic Fractures in Men Study. J. Bone Miner. Res..

[30] Sheu Y., Cauley J.A., Bunker C.H., Wheeler V.W., Patrick A.L., Gordon C.L., Kammerer C.M., Zmuda J.M. (2009). Correlates of trabecular and cortical volumetric BMD in men of African ancestry. J. Bone Miner. Res..

[31] Burr D.B. (1993). Remodeling and the repair of fatigue damage. Calcif. Tissue Int..

[32] Lorentzon M., Landin K., Mellström D., Ohlsson C. (2006). Leptin is a negative independent predictor of areal BMD and cortical bone size in young adult Swedish men. J. Bone Miner. Res..

[33] Warensjo E., Byberg L., Melhus H., Gedeborg R., Mallmin H., Wolk A., Michaelsson K. (2011). Dietary calcium intake and risk of fracture and osteoporosis: Prospective longitudinal cohort study. BMJ.

[34] Kim K.M., Choi S.H., Lim S., Moon J.H., Kim J.H., Kim S.W., Jang H.C., Shin C.S. (2014). Interactions between dietary calcium intake and bone mineral density or bone geometry in a low calcium intake population (KNHANES IV 2008–2010). J. Clin. Endocr. Metab..

[35] Bischoff-Ferrari H.A., Kiel D.P., Dawson-Hughes B., Orav J.E., Li R., Spiegelman D., Dietrich T., Willett W.C. (2009). Dietary calcium and serum 25-hydroxyvitamin D status in relation to BMD among US adults. J. Bone Miner. Res..

[36] Mezquita-Raya P., Munoz-Torres M., Luna J.D., Luna V., Lopez-Rodriguez F., Torres-Vela E., Escobar-Jimenez F. (2001). Relation between vitamin D insufficiency, bone density, and bone metabolism in healthy postmenopausal women. J. Bone Miner. Res..

[37] POF (2011). Pesquisa de orçamentos familiares 2008–2009. Análise do Consumo Alimentar Pessoal no Brasil.

[38] Lips P. (2001). Vitamin D deficiency and secondary hyperparathyroidism in the elderly: Consequences for bone loss and fractures and therapeutic implications. Endocr. Rev..

[39] Bouillon R., Van Schoor N.M., Gielen E., Boonen S., Mathieu C., Vanderschueren D., Lips P. (2013). Optimal vitamin D status: A critical analysis on the basis of evidence-based medicine. J. Clin. Endocr. Metab..

[40] Bischof Kota S., Jammula S., Kota S., Meher L., Modi K. (2013). Correlation of vitamin D, bone mineral density and parathyroid hormone levels in adults with low bone density. Indian J. Orthop..

[41] Sahota O., Mundey M.K., San P., Godber I.M., Lawson N., Hosking D.J. (2004). The relationship between vitamin D and parathyroid hormone: Calcium homeostasis, bone turnover, and bone mineral density in postmenopausal women with established osteoporosis. Bone.

[42] Iuliano-Burns S., Ayton J., Hillam S., Jones G., King K., Macleod S., Seeman E. (2012). Skeletal and hormonal responses to vitamin D supplementation during sunlight deprivation in antarctic expeditioners. Osteoporosis. Int..

[43] Villareal D.T., Civitelli R., Chines A., Avioli L.V. (1991). Subclinical vitamin-d deficiency in postmenopausal women with low vertebral bone mass. J. Clin. Endocr. Metab..

[44] Bischoff-Ferrari H.A., Dietrich T., Orav E.J., Dawson-Hughes B. (2004). Positive association between 25-hydroxy, vitamin D levels and bone mineral density: A population-based study of younger and older adults. Am. J. Med..

[45] Smith L.M., Gallagher J.C., Kaufmann M., Jones G. (2018). Effect of increasing doses of vitamin D on bone mineral density and serum n-terminal telopeptide in elderly women: A randomized controlled trial. J. Intern. Med..

[46] Gannage-Yared M.H., Chemali R., Yaacoub N., Halaby G. (2000). Hypovitaminosis d in a sunny country: Relation to lifestyle and bone markers. J. Bone Miner. Res..

[47] Olmos J.M., Hernandez J.L., Garcia-Velasco P., Martinez J., Llorca J., Gonzalez-Macias J. (2016). Serum 25-hydroxyvitamin d, parathyroid hormone, calcium intake, and bone mineral density in Spanish adults. Osteoporos. Int..

[48] Liu X.S., Cohen A., Shane E., Yin P.T., Stein E.M., Rogers H., Kokolus S.L., McMahon D.J., Lappe J.M., Recker R.R. (2010). Bone Density, Geometry, Microstructure and Stiffness: Relationships Between Peripheral and Central Skeletal Sites Assessed by DXA, HR-pQCT, and cQCT in Premenopausal Women. J. Bone Miner. Res..

