# Supplementary material for: Association between 25-Hydroxyvitamin D, Parathyroid Hormone, Vitamin D and Calcium Intake, and Bone Density in Healthy Adult Women: A Cross-Sectional Analysis from the D-SOL Study
Source: Nutrients. 2019 Jun 4;11(6):1267. doi: 10.3390/nu11061267 (PMC6628165; doi:10.3390/nu11061267)
Supplement: Supplementary file 1 [file nutrients-11-01267-s001.pdf]

Table S1. Characteristics of Brazilian women living in England by tertile of 4% Total vBMD (n=51)[1]

Bone Parameter	4% Total vBMD (mg/cm3) Tertile 1 < 327.31	Tertile 2 327.31 – 365.51	Tertile 3 > 365.51	p value[2]
Number (n)	17	17	17	
**Wegiht (kg)**	67.17 ±9.5	73.60 ±13.56	69.54 ±18.30	0.419
**Height (m)**	1.64 ±0.05	1.64 ±0.06	1.64 ±0.05	0.912
**BMI (kg/m²)**	24.92 ±4.11	27.08 ±4.91	25.84 ±6.65	0.500
**Serum 25(OH)D (nmol/l)**	37.44 ±14.74	38.01 ±17.22	30.20 ±11.80	0.238
**Plasma PTH (pmol/l)**	5.38 ±1.57	4.94 ±2.73	5.94 ±2.73	0.364
**Serum calcium (mmol/l)**	2.29 ±0.06	2.31 ±0.08	2.28 ±0.07	0.463

[1]Values: mean ± SD; [2] Statistical analysis: one-way ANOVA with post-hoc Tukey's test.

Table S2. Characteristics of Brazilian women living in England by tertile of 4% Trabecular vBMD (n=51)[1]

Bone Parameter	4% Trabecular vBMD (mg/cm3) Tertile 1 < 172.59	Tertile 2 172.59 – 202.81	Tertile 3 > 202.81	p value[2]
Number (n)	17	16	17	
**Wegiht (kg)**	73.10 ±14.83	65.44 ±11.35	72.26 ±15.63	0.246
**Height (m)**	1.67 ±0.051[a]	1.62 ±0.04[a]	1.63 ±0.06	**0.008**
**BMI (kg/m²)**	26.09 ±5.61	24.92 ±4.31	26.96 ±6.01	0.555
**Serum 25(OH)D (nmol/l)**	37.14 ±17.09	34.02 ±11.71	34.84 ±16.25	0.829
**Plasma PTH (pmol/l)**	5.45 ±2.38	5.48 ±1.65	5.40 ±2.16	0.994
**Serum calcium (mmol/l)**	2.30 ±0.06	2.32 ±0.06	2.26 ±0.09	0.052

[1]Values: mean ± SD; [2] Statistical analysis: one-way ANOVA with post-hoc Tukey's test: values in same row with same superscript letters are significantly different ([a] *p*=0.007).

Table S3. Characteristics of Brazilian women living in England by tertile of 66% Cortical vBMD (n=51)[1]

Bone Parameter	66% Cortical vBMD (mg/cm3) Tertile 1 < 1113.00	Tertile 2 1113.00 – 1149.48	Tertile 3 > 1149.48	p value[2]
Number (n)	17	17	17	
**Wegiht (kg)**	74.21 ±16.86	71.95 ±11.41	64.16 ±12.56	0.095
**Height (m)**	1.64 ±0.05	1.63 ±0.06	1.65 ±0.05	0.732
**BMI (kg/m²)**	27.36 ±6.02	26.86 ±3.98	23.61 ±5.19	0.079

**Serum 25(OH)D (nmol/l)**	34.40 ±18.02	37.85 ±15.03	33.41 ±11.47	0.667
**Plasma PTH (pmol/l)**	5.69 ±2.89	5.21 ±1.44	5.26 ±1.67	0.762
**Serum calcium (mmol/l)**	2.30 ±0.06	2.30 ±0.70	2.27 ±0.07	0.380

[1]Values: mean ± SD; [2] Statistical analysis: one-way ANOVA with post-hoc Tukey's test.

Table S4. Characteristics of Brazilian women living in England by tertile of 66% Total vBMD (n=51)[1]

Bone Parameter	66% Total vBMD (mg/cm3)			p value[2]
	Tertile 1	Tertile 2	Tertile 3	
	< 676.04	676.04 – 749.19	> 749.19	
Number (n)	17	17	17	
**Wegiht (kg)**	75.14 ±17.33	69.40 ±10.41	66.78 ±13.46	0.194
**Height (m)**	1.65 ±0.06	1.63 ±0.04	1.64 ±0.06	0.687
**BMI (kg/m²)**	27.55 ±6.16	25.54 ±3.58	24.74 ±5.70	0.286
**Serum 25(OH)D (nmol/l)**	28.43 ±12.86	41.03 ±15.77	36.21 ±17.91	0.402
**Plasma PTH (pmol/l)**	6.10 ±2.71	4.98 ±1.63	5.08 ±1.61	0.225
**Serum calcium (mmol/l)**	2.28 ±0.06	2.33 ±0.73	2.28 ±0.08	0.094

[1]Values: mean ± SD; [2] Statistical analysis: one-way ANOVA with post-hoc Tukey's test.

Table S5. Characteristics of Brazilian women living in Brazil by tertile of lumbar spine BMD (n=79)[1]

Bone Parameter	Lumbar spine BMD (g/cm²)			p value[2]
	Tertile 1	Tertile 2	Tertile 3	
	< 1.077	1.077 – 1.185	> 1.185	
Number (n)	26	27	26	
**Wegiht (kg)**	58.11 ±10.95[a]	64.21 ±14.80	67.27 ±11.82[a]	**0.034**
**Height (m)**	1.61 ±0.06	1.62 ±0.05	1.63 ±0.06	0.300
**BMI (kg/m²)**	22.40 ±4.52	24.41 ±5.23	25.22 ±4.57	0.099
**Serum 25(OH)D (nmol/l)**	73.52 ±21.26	76.71 ±24.02	74.71 ±21.78	0.872
**Plasma PTH (pmol/l)**	4.47 ±1.44	4.45 ±1.58	4.54 ±1.45	0.973
**Serum calcium (mmol/l)**	2.26 ±0.05	2.29 ±0.06	2.28 ±0.06	0.102

[1]Values: mean ± SD; [2] Statistical analysis: one-way ANOVA with post-hoc Tukey's test: values in same row with same superscript letters are significantly different ([a] *p*=0.029).

Table S6. Characteristics of Brazilian women living in Brazil by tertile of femur BMD (n=64)[1]

Bone Parameter	Femur BMD (g/cm²)			p value[2]
	Tertile 1	Tertile 2	Tertile 3	
	< 0.931	0.931 – 1.026	> 1.026	

Number (n)	22	21	21	
**Wegiht (kg)**	56.85 ±8.03[a]	64.49 ±10.89[b]	73.79 ±15.40[a,b]	**>0.001**
**Height (m)**	1.62 ±0.07	1.61 ±0.06	1.61 ±0.05	0.810
**BMI (kg/m²)**	21.45 ±2.67[c,d]	24.69 ±3.88[c,e]	28.26 ±5.70[d,e]	**>0.001**
**Serum 25(OH)D (nmol/l)**	72.85 ±18.40	74.26 ±26.42	73.16 ±16.83	0.974
**Plasma PTH (pmol/l)**	4.43 ±1.23	4.21 ±1.23	5.15 ±1.92	0.115
**Serum calcium (mmol/l)**	2.27 ±0.05	2.32 ±0.06	2.27 ±0.05	0.800

[1] Values: mean ± SD; [2] Statistical analysis: one-way ANOVA with post-hoc Tukey's test: values in same row with same superscript letters are significantly different ([a] p= >0.001; [b] p=0.035; [c] p=0.040; [d] p<0.001; [e] p=0.023
